# Burden of paediatric Rotavirus Gastroenteritis (RVGE) and potential benefits of a universal Rotavirus vaccination programme with a pentavalent vaccine in Spain

**DOI:** 10.1186/1471-2458-10-469

**Published:** 2010-08-10

**Authors:** Javier Diez-Domingo, Nuria Lara Suriñach, Natalia Malé Alcalde, Lourdes Betegón, Nathalie Largeron, Mélanie Trichard

**Affiliations:** 1 Vaccine Investigation Area. CSISP Centre for Public Health Research, Avda Catalunya 21, Valencia, (46020), Spain; 2 HEOR, IMS Health, Dr. Ferran 25 - 27, Barcelona, (08034), Spain; 3 Market Access Manager Sanofi PasteurMSD, SNC, 8, Rue Jonas Salk, Lyon,(69367), France

## Abstract

**Background:**

Rotavirus is the most common cause of gastroenteritis in young children worldwide. The aim of the study was to assess the health outcomes and the economic impact of a universal rotavirus vaccination programme with RotaTeq, the pentavalent rotavirus vaccine, versus no vaccination programme in Spain.

**Methods:**

A birth cohort was followed up to the age of 5 using a cohort model. Epidemiological parameters were taken from the REVEAL study (a prospective epidemiological study conducted in Spain, 2004-2005) and from the literature. Direct and indirect costs were assessed from the national healthcare payer and societal perspectives by combining health care resource utilisation collected in REVEAL study and unit costs from official sources. RotaTeq per protocol efficacy data was taken from a large worldwide rotavirus clinical trial (70,000 children). Health outcomes included home care cases, General Practioner (GP)/Paediatrician, emergency department visits, hospitalisations and nosocomial infections.

**Results:**

The model estimates that the introduction of a universal rotavirus vaccination programme with RotaTeq (90% coverage rate) would reduce the rotavirus gastroenteritis (RVGE) burden by 75% in Spain; 53,692 home care cases, 35,187 GP/Paediatrician visits, 34,287 emergency department visits, 10,987 hospitalisations and 2,053 nosocomial infections would be avoided. The introduction of RotaTeq would avoid about 76% of RVGE-related costs from both perspectives: €22 million from the national health system perspective and €38 million from the societal perspective.

**Conclusions:**

A rotavirus vaccination programme with RotaTeq would reduce significantly the important medical and economic burden of RVGE in Spain.

## Background

Acute Gastroenteritis (AGE) is a common disease among children in both developed and developing countries, with rotavirus as the principal etiologic agent[1,2]. As it is a highly contagious virus, almost all children will suffer from paediatric rotavirus gastroenteritis (RVGE) before 5 years of age[3]. Verstraeten et al[4] estimated that 4.5 million episodes of RVGE occur each year in the European Union among children up to 5 years old. The classical symptoms of the disease are diarrhoea, vomiting and fever[5].

Although death due to RVGE is rare in developed countries, there is an important morbidity related to the disease as well as a substantial economic burden associated with its management[6]. RVGE is a major reason for hospitalisation, and it is responsible for an important number of nosocomial infections in paediatric wards, which increase the medical resources required for treating these children with RVGE[7,8]. According to Gil et al[3], the annual incidence of hospital admissions attributable to rotavirus is 1.0 per 1000 children ≤5 years, although it can be as high as 2.5 per 1000 children ≤5 years during the winter season. Overall, these authors estimated that the annual number of days of hospitalisation attributable to rotavirus exceeds 8,700 in Spain. Another important consequence of the disease is the workdays lost by parents and caregivers[9,10]. This has been estimated to account for 75% of total costs of RVGE in a primary care setting in Italy[11]. Recent publications in different European countries have added information about the burden and costs of the disease as well as the potential benefits of a universal vaccination programme[9,12-19].

An oral, pentavalent rotavirus vaccine (RotaTeq), which has recently been licensed in Europe, has been shown to be highly efficacious and safe in a large-scale phase III trial (Rotavirus Efficacy Safety Trial; REST)[20]. This vaccine offers protection against G1, G2, G3, G4 and P[8] serotypes, which are responsible for 98% of all RVGE episodes[21] in Europe, and is available in Spain. Several published studies have suggested that effective rotavirus immunisation would provide large health and economic benefits in Europe[22,23]; however, no specific data about the potential benefits of a universal rotavirus vaccination programme in Spain were available until now. The objective of this study was to assess the potential health and economic benefits of a universal vaccination with a pentavalent rotavirus vaccine (RotaTeq) from the National Health System (NHS) and societal perspectives in the Spanish setting using a modelling approach. The present study is not a cost-effectiveness analysis.

## Methods

### Model design

**Figure 1:**
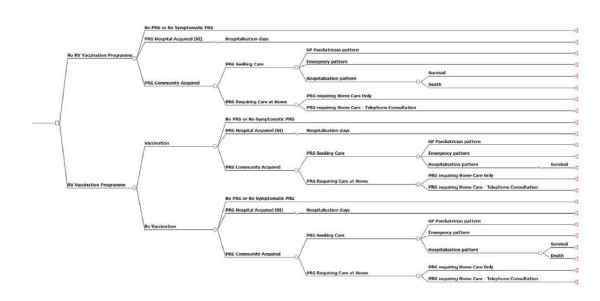
**Model tree overview - Rotavirus vaccination program versus no Rotavirus vaccination program.** PRG: Paediatric Rotavirus Gastroenteritis (RVGE)

A health-economic cohort model was developed to assess the costs and health benefits of a universal rotavirus vaccination programme with RotaTeq versus no rotavirus vaccination programme by simulating the flow of a hypothetical cohort of infants from birth to 5 years. Under each scenario, children may experience or not a RVGE episode, which may be either community or hospital acquired. When community cases occurred, patients may seek medical attention either at a primary care centre (PCC), at an emergency room (ER) or at hospital. Also, patients may not seek medical attention and only require home care. Hospital acquired episodes (nosocomial infections) were assumed to require additional days of hospitalisation. The "vaccination strategy" arm included children receiving and not receiving the vaccine with a coverage rate of 90%[24]. The structure of the decision model is shown in Figure 1.

Based on incidence data, each children was at risk of RVGE with corresponding resource consumption and costs, which were obtained from different sources: the Rotavirus Gastroenteritis Epidemiology and Viral Types Accounting for Losses in Public Health and Society (REVEAL) epidemiological study[25], the REVEAL costs study[26], the Rotavirus Efficacy & Safety Trial (REST)[20], national statistics[27], and from an extensive literature review[28-35]. A discount rate of 3% was applied to both costs and benefits based on results from recent Spanish pharmacoeconomic studies[36,37].

### Model inputs

#### Epidemiological data

Epidemiological data for patients seeking medical attention were obtained from the REVEAL study[25], which was the first European-wide epidemiological study about rotavirus, specifically conducted to measure epidemiological consequences and the burden of RVGE. This prospective, multicenter, observational study on children up to 5 years old with AGE was performed between October 2004 and September 2005 in seven European countries including Spain. In Spain, two hospitals, three ERs and twenty-three PCCs participated in the study and a total of 801 children were included. A total of 252 of the 772 stool samples analysed for the presence of rotavirus, by an ELISA test, were positive (32.6%). A questionnaire was completed for each child included to collect data on resource utilisation (consultations, hospitalisations, drug consumption, clinical services, extra childcare, transportation and additional nappies) and number of parental workdays lost. These data were used to estimate annual incidence rates of RVGE consultations at hospital, ER and PCC, and to evaluate the impact of RVGE on the health care system (health care consumption) and the society (productivity loss).

Based on the REVEAL study[25], the annual incidence rates for hospitalisations, ER and PCC consultations in patients with RVGE seeking medical attention were 6.5/1,000, 18.9/1,000 and 21.9/1,000, respectively. Age-specific incidences were calculated for seven age groups (0-3 months, 3-6 months, 6-12 months, 1-2 years, 2-3 years, 3-4 years and 4-5 years) by deriving annual incidences with the specific age group distribution obtained from REVEAL study.

The proportion of children with RVGE who did not seek medical care was estimated to be 41.4%. As such data is not available for Spain; this estimate was taken from the incidence observed in a study preformed in a daycare centre in France (Lyon)[33].

The age group distribution for children who did not seek medical care was assumed to be similar to the one observed in children visiting a PCC estimated in the REVEAL study[25].

The incidence of nosocomial infection in children up to 5 years used in the model was 1.6/1,000[29]. The age group distribution was derived from the percentages reported from Gleizes et al[29] and Forster et al[38]. The average extra length of stay due to nosocomial infections was assumed to be 2.4 days based on the average results from two Spanish publications[30,31].

According to the Spanish National Statistics Institute for 2004 (INE)[27], the annual number of births in Spain was 441,283 and life expectancy was 78.7 years (average for males and females, year 1998). UNICEF[28] has reported a mortality rate for children under 5 years in Spain of 4/1,000. The epidemiological parameters for the model are shown in Table 1.

**Table 1:** Data for demographics, epidemiological and parental work days lost

Model parameters	Value	References
Demographics		
Birth cohort size	441,283	23
Life expectancy	78.7 years	23
Mortality rate in case of no vaccination < 5 years	4/1,000	24
% of cases not seeking medical care	41.4%	29
% of G1-G2-G3-G4	97%	17

RVGE Burden (< 5 years)		
Annual hospitalization incidence rate	6.5/1000	21
Age group distribution (%):		
< 3 months	7.69	
3-6 months	5.77	
6-12 months	17.31	
12-24 months	34.62	
24-36 months	21.15	
36-48 months	9.62	
48-60 months	3.84	
Annual emergency visits incidence rate	18.9/1000	21
Age group distribution (%):		
< 3 months	1.0	
3-6 months	16.0	
6-12 months	22.0	
12-24 months	46.0	
24-36 months	11.0	
36-48 months	3.0	
48-60 months	1.0	
Annual PCC visits incidence rate	21.9/1000	21
Age group distribution (%):		
< 3 months	3.06	
3-6 months	6.12	
6-12 months	22.45	
12-24 months	39.80	
24-36 months	19.39	
36-48 months	6.12	
48-60 months	3.06	
Annual nosocomial infection incidence rate	1.6/1000	25
Age group distribution (%):		
< 3 months	31.85	
3-6 months	16.15	
6-12 months	26	
12-24 months	19	
24-36 months	5.5	
36-48 months	1.5	
48-60 months	0	

Parental work days lost (mean number of days/paediatric case)		
Hospitalized RVGE case	3.12	21
RVGE case seen in emergency visit	2.02	21
RVGE case seen in PCC visit	1.37	21
RVGE nosocomial infection	1.63	Assumption based on 21, 26, 27
RVGE not seeking medical attention	0.26	Assumption based on 37

#### Vaccine parameters

The efficacy and safety of the pentavalent rotavirus vaccine (RotaTeq) have been demonstrated in the REST trial[20], a large-scale, double-blind, placebo controlled, randomised international trial conducted from 2001 to 2004 in 11 different countries which included 70,301 healthy infants aged between 6 and 12 weeks. Children received three doses of the oral vaccine or placebo, at 2, 4 and 6 months, with 4 to 10 weeks intervals between doses.

Per-protocol efficacy data against G1-G4 RVGE cases was used in the model. Based on REST data, a reduction of 95.8%, 93.7% and 86.0% in the incidence of hospitalisations, ER, and GP visits respectively was assumed for all vaccinated children. Although REST data specifically relates to G1-G4 RVGE, the reduction in health care utilisation due to serotypes other than G1-G4 was considered to be the same as for vaccine-contained serotypes[16]. RotaTeq also contains P[8], a predominant genotype and the most common P-type associated with human RV strains in Europe and worldwide. Studies suggest vaccines containing the serotype P[8] may protect against other G-serotypes, such as G9, which was confirmed for RotaTeq in the REST study. G1, G2, G3, G4 and G9 serotypes account for 98% of RVGE cases in Spain[21].

As it was not specifically evaluated in the clinical study, it was assumed that the vaccine efficacy for nosocomial infections was similar to that observed for hospitalisation, and that for patients not seeking medical care was similar to that observed for PCC visits.

Vaccine efficacy was considered to be nil in the 0-3 months age group, and full protection started from the 3-6 months age group.

The model took into account the waning immunity observed in REST[20], which was a 10% decrease in vaccine efficacy 2 years post-vaccination. The vaccine coverage rate was assumed to be 90%, based on the observed coverage for common routine vaccinations in Spain (diphtheria, tetanus and pertussis)[24]. The vaccine parameters used in the model are shown in Table 2.

**Table 2:** Vaccination parameters

Parameters	Value	Source
Coverage rate	90%	20
Vaccine efficacy (%):		
Hospitalizations reduction	95.80	16
Emergency visits reduction	93.70	16
PCC visits reduction	86.00	16
Nosocomial infections reduction	95.80	Assumption: same as hospitalization
Cases not seeking care reduction	86.00	Assumption: same as PCC
		
Waning rate after 2 years	10%	Based on REST (16) and expert opinions

#### Cost data

Costs were assessed from both NHS and societal perspectives. Costs for children who were hospitalised, or who had ER and PCC visits were obtained from the Spanish REVEAL study of costs[26]. Cost of nosocomial infections was calculated based on an estimated extra length of stay of 2.4 days and the cost per day of hospitalisation of €310[31]. It was assumed that 68.3% of parents had to take time off work for children who were hospitalised[25]. Costs for children not seeking medical care were considered from the societal perspective. It was estimated that an average of 0.26 workdays were lost per episode not involving medical care, based on the assumption that mothers would stop working half a day, and taking into account that 51% of mothers work outside their home in Spain[39]. Unit costs from local sources[40-45] were used to value each resource. The cost parameters used in the model are presented at Table 3.

**Table 3:** Cost per RVGE cases

Parameters	NHS perspective	Societal perspective	Source
RVGE cases not seeking care	0 €	23.80€	Assumption based on 37, 42

Hospitalized RVGE cases	1,248.99€	1,551.70€	22

Emergency visit RVGE cases	204.29€	408.87€	22

PCC RVGE cases	16.61€	165.89€	22

Nosocomial infection cost	744 €	896.80€	Assumption based on 26, 27, 42

##### - Direct medical costs

According to each healthcare setting, direct medical costs for consultations, hospitalisations including, medication and laboratory tests were considered. Hospitalisation costs were calculated using DRG data (Diagnostic-Related Groups).

In Spain, the cost of consultations, hospitalisations and laboratory tests are fully covered by the NHS. The prescribed medication costs are shared between the patients and the NHS based on a fixed percentage (40% for the patients and 60% for the NHS). Over-The-counter (OTC) drugs are not reimbursed by the NHS.

##### - Non-medical costs

The non-medical costs included transportation (car or taxi), extra nappies and baby sitting; which were extracted from the REVEAL study[26]. These costs are paid by the patients.

##### - Indirect costs

Work days lost by parents seeking medical care for their child was estimated at 3.12 days for hospitalisation, 2.02 days for emergency room visits and 1.37 days for PCC offices visits[26]. For those who did not seek medical care the number of workdays lost was estimated to be 0.26 days based on the assumptions that 51% of mothers work outside the home[39] and that they stopped working for half a day to take care of their children. The cost for one workday lost was 93.3€ based on Eurostat data[44]. Indirect costs were included in the societal perspective.

### Model outcomes

The model was developed to compare the health outcomes (number of RVGE cases whether or not seeking medical care, number of hospitalisations, number of ER and PCC visits and number of nosocomial infections) and costs from NHS and societal perspectives for the vaccination and not vaccination scenario.

The number needed to vaccinate (NNV) for Spain was calculated using data from the health economic model for a vaccinated birth cohort. This gives the number of children to vaccinate to avoid one child seeking medical care (hospitalisation, ER visit, PCC) due to RVGE. The NNV was calculated following Brisson et al [13] method which assessed the NNV to prevent Human PapillomaVirus (HPV) related diseases. It is defined as the reciprocal of the percentage of children seeking medical care in the group without rotavirus vaccination minus the percentage in the group with rotavirus vaccination.

#### - Sensitivity analyses

Since some parameters may present uncertainty, a one-way deterministic sensitivity analyses was performed, using 95% confidence intervals (CI) when available, data from the literature or varying the base case value with a fixed percentage (± 20%) when no other source could be considered. The variables that were tested were the vaccine coverage rate, the vaccine efficacy in reduction of RVGE cases, the vaccine efficacy annual rate by age, epidemiological data, costs of the disease and discount rates (Table 4).

**Table 4:** Tested parameters for the sensitivity analyses

Parameters	Base case	Sensitivity analyses	Source for the sensitivity analyses
**Vaccine coverage rate**	90%	50%/97%	Ref 50
**Ratio non seeking medical care/seeking medical care**	70.65%	56.52%/84.78%	± 20%
**Discount rates:**			
For costs	3%	6%/0%	Assumption
For benefits	3%	6%/0%	Assumption
**Incidences:**			
PCC	2.19%	1.752%/2.628%	± 20%
Hospitalization	0.65%	0.52%/0.78%	± 20%
Emergency visits	1.89%	1.512%/2.268%	± 20%
Nosocomial infections	0.16%	0.128%/0.192%	± 20%
**Vaccine efficacy in reduction of RVGE cases:**			
PCC visits	86%	73.9%/92.5%	95% CI
Emergency visits	93.7%	88.8%/96.5%	95% CI
Hospitalization	95.8%	90.5%/98.2%	95% CI
**Vaccine efficacy annual rate by year**	Constant decrease by year of 10%	Exponential decrease after year 2.	Assumption
**Cost of the disease, NHS perspective:**			
PCC visit	16.61€	14.62€/18.63€	95% CI
Emergency visit	204.29€	186.19€/222.39€	95% CI
Hospitalization among non vaccinated	1248.99€	1110.35€/1386.38€	95% CI
Nosocomial infection	744.00€	706.80€/781.20€	95% CI
**Cost of the disease, societal perspective:**			
PCC visit	165.89€	110.81€/221.05€	95% CI
Emergency visit	408.87€	330.65€/486.96€	95% CI
Hospitalization among non vaccinated	1551.70€	1364.72€/1738.68€	95% CI
Nosocomial infection	896.80€	851.96€/941.64€	95% CI

## Results

### Burden and costs of RVGE in Spain

The model estimated that each year, 181,626 children would have an RVGE episode, resulting in 14,342 hospitalisations, 41,701 visits to an ER, 48,320 visits to a PCC, 3,530 nosocomial infections and 210,404 work days lost for the parents. In addition, 73,733 cases would not seek medical care.

The annual costs due to RVGE in children under 5 years old were estimated to be €28.6 million from the NHS perspective, while, from the societal perspective they were estimated to be €50.0 million.

### Expected health and economic benefits from a universal rotavirus vaccination programme

The implementation of a 3-dose universal rotavirus vaccination programme with RotaTeq would have a positive impact on public health in Spain since, assuming a 90% coverage rate, the programme would prevent 136,190 episodes of RVGE annually. This would lead to the prevention of 10,981 hospitalisations (-76%), 34,287 ER visits (-82%), 35,187 consultations with a PCC (-73%), 2,053 nosocomial infections (-58%) and 53,692 RVGE episodes for which no medical care is sought (-73%). Furthermore, the programme would avoid 161,495 work days being lost (-77%) (Table 5).

**Table 5:** Base case health outcomes under the current context and under a universal rotavirus vaccination program for a birth cohort followed until 5 years

	No rotavirus vaccination program	With rotavirus vaccination program	Avoided cases with rotavirus vaccination program	Reduction (%)
**RVGE cases seeking medical care**	**107,894**	**25,380**	**82,514**	**-76.5%**
Hospitalizations	14,342	3,355	10,987	-76%
Emergency visits	41,701	7,414	34,287	-82%
PCC visits	48,320	13,133	35,187	-73%
Nosocomial infections	3,530	1,477	2,053	-58%
**RVGE cases not seeking medical care**	73,733	20,040	53,692	-73%
**Total RVGE cases**	181,626	45,420	136,206	-75%
**Parental work days lost**	210,404	48,909	161,495	-77%

**Figure 2:**
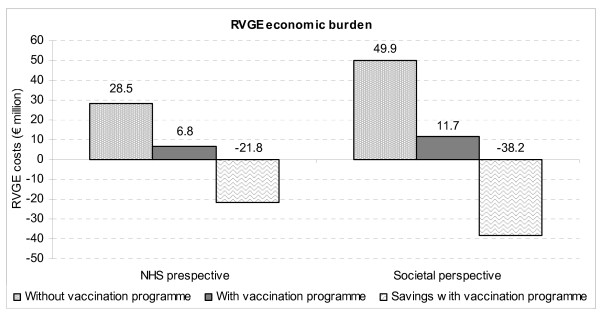
RVGE economic burden in Spain with and without a universal rotavirus vaccination program.

The model predicted that for every 5 infants vaccinated with RotaTeq, one need for medical care for RVGE (hospitalisation, ER visit, PCC) would be avoided. Based on the benefits of vaccination, the model predicted that the total costs associated with RVGE in a Spanish birth cohort followed up to 5 years of age would be reduced by €22 million for the NHS (-76.3%) and lead to an overall reduction of €38 million from the societal perspective (-76.5%) (Figure 2).

### Sensitivity analyses

Health and economic outcomes were most sensitive to changes in the vaccine coverage rate and health care setting incidences. Benefits of a universal rotavirus vaccination programme would be increased if the vaccine coverage was higher, which is an expected finding in the health economic evaluations of vaccines. The same effect would be observed with the increase in the rotavirus incidence at any setting, the vaccine efficacy and the costs.

Hospitalisation incidence was tested based on an assumed 20% variation. For the higher value, the overall number of avoided paediatric rotavirus hospitalisations would be increased in comparison to the base case from 10,981 to 13,184 and consequently the overall avoided paediatric RVGE costs from €22 to €25 million and from €39 to €42 million from the NHS and the societal perspectives, respectively. Work days lost by parents were more sensitive to the variations in the incidence of emergency visits than to variations in hospitalisations incidence; a 20% increase in emergency visits would result in 14,376 extra days lost in comparison to the base case. The results of the sensitivity analyses are shown in Table 6.

**Table 6:** Results of the sensitivity analyses

	Number of events avoided						RVGE costs avoided (M€)	
	RVGE cases (seeking or nor medical care)	Hospitali-zations	Nosocomial infections	Emergency visits	PCC visits	Work Days Lost	NHS perspec-tive	Societal perspec-tive
**BASE CASE**	136,190	10,987	2,053	34,287	35,187	161,495	21.77	38.19
**SENSITIVITY ANALYSES**								
**Vaccine coverage rate**								
50%	75,670	6,104	1,141	19,049	19,548	89,719	12.09	21.20
97%	146,800	11,841	2,213	36,954	37,924	174,055	23.47	41.16
**Ratio not seeking medical care/seeking medical care**								
-20%	125,468	10,987	2,053	34,287	35,187	158,836	21.77	37.95
+20%	146,945	10,987	2,053	34,287	35,187	164,415	21.77	38.43
**Cost and benefit discount rates**								
0%; 0%	136,207	10,987	2,053	34,287	35,187	169,052	22.84	40.02
6%; 6%	136,207	10,987	2,053	34,287	35,187	154,371	20.77	36.46
**Incidences**								
**PCC**								
-20%	124,197	10,987	2,053	34,287	28,150	151,082	21.66	36.96
+20%	148,216	10,987	2,053	34,287	42,225	171,908	21.88	39.41
**Hospitalization**								
-20%	132,534	8,789	2,053	34,287	35,187	154,633	19.17	34.92
+20%	139,880	13,184	2,053	34,287	35,187	168,357	24.37	41.45
**Emergency visits**								
-20%	125,058	10,987	2,053	27,430	35,187	147,119	20.43	35.40
+20%	147,355	10,987	2,053	41,145	35,187	175,870	23.12	40.98
**Nosocomial infections**								
-20%	135,796	10,987	1,642	34,287	35,187	160,846	21.48	37.83
+20%	136,617	10,987	2,464	34,287	35,187	162,143	22.07	38.55
**Vaccine efficacy in reduction of RVGE cases (95% CI)**								
90.5%: hospitalizations, NI 88.8%: emergency visits 73.9%: PCC and home care	121,187	10,379	1,939	32,494	30,236	147,707	20.54	35.54
98.2%: hospitalizations, NI 96.5%: emergency visits 92.5%: PCC and home care	144,258	11,262	2,104	35,312	37,847	168,854	22.39	39.55
**Vaccine efficacy annual rate by age**								
10% decrease in year 2 and exponential decrease until year 4.	135.507	10,891	2,051	34,208	34,987	160,770	21.65	37.99
**NHS & Societal costs (95% CI)**								
Lower values	136,1901	10,987	2,053	34,287	35,187	161,4951	19.59	33.57
Higher values	36,190	10,987	2,053	34,287	35,187	61,495	23.94	44.64

## Discussion

AGE is a frequent disease, with rotavirus being the main cause in children under 5 years of age. The burden of RVGE is considerable both in terms of clinical and economic considerations[6,46,47]. To our knowledge this is the first study to estimate the burden of paediatric RVGE, the associated direct and indirect costs, and the potential health and economic benefits of a universal rotavirus vaccination programme in Spain. The model predicted that RVGE would be responsible for more than 181,600 infections for every new birth cohort in Spain followed up to 5 years of age and would result in intensive use of health care services as well as more than 210,000 work days lost by parents. The implementation of a universal vaccination programme for infants in Spain could potentially reduce the overall clinical burden of RVGE by 75% and save 76% of costs from both the NHS and the societal perspectives.

We estimate that RVGE is responsible for more than 14,000 hospitalisations a year in children up to 5 years old in Spain. This incidence is higher than that previously reported, which was 9,000 days of hospitalisation a year in children of the same age group[3]. The difference might be explained by the methodology used since in the previous study hospitalisation for RVGE was estimated using data on laboratory reports and hospital admissions due to AGE. Our data were based on the results from a recent prospective epidemiological and cost-of-illness study (REVEAL)[25], which included all children aged up to 5 years old with RVGE over a one year period in one Spanish region, so our estimation might reflect the current burden of hospitalisations due to RVGE in Spain.

The annual incidence rates for PCC consultations in patients with RVGE seeking medical attention assumed in this study based on REVEAL, is very similar to the results in other previous studies performed in Spain[48,35].

Furthermore a recent review of prospective studies suggested that the total burden of symptomatic rotavirus infection does not differ significantly between studies in different countries around the world, even though use of health care by infected patients does [49].

A recent study[50] in the Galician area of Spain estimated that the mean indirect cost per case due to RVGE was €428. This cost included productivity loss, travel expenses, cost of caregivers, meals and materials. Although our definition of indirect costs referred only to productivity loss, our model included transportation, extra nappies and baby sitting in terms of non-medical costs. Taking these costs together, our estimated cost is lower, ranging form €139 to €289 (PCC and hospital cases, respectively). The work days lost by parents accounted for 69% and 75% of the overall cost in studies conducted in the United States[51] and in Italy[11], respectively, whereas in our model this was only 39% of the overall cost of €19.6 million. This suggests that our analysis might underestimate the real burden of RVGE in Spain.

Although there are no other studies evaluating the impact of a universal rotavirus vaccination in Spain, our findings are consistent with studies performed in France[12], UK[52] and Germany[13], that estimated that a universal vaccination programme, with a 90% coverage, would reduce the burden of disease by 74% to 75%.

The main objective of the study was to assess the economic burden of rotavirus disease and to describe the potential benefits of rotavirus vaccination, but not its cost-effectiveness. A cost effectiveness analysis is usually used as a decision making tool for resource allocation in a situation of limited resources. In order to perform a cost effectiveness analysis, a reliable vaccine price should be considered. However, the current situation in Spain is that the vaccine is currently available on an out-of-pocket market, i.e., not reimbursed by the national health system with different prices depending on the region. It is consequently difficult to assess the current market price. Also, the expected tender price under reimbursement from national health system would be lower than the current market price. Therefore, using the current market price for the vaccine would substantially unfavour the cost-effectiveness results, and even more, results would not be reliable because of the use of un real vaccine price.

However, a cost-effectiveness analysis would be of interest using various price assumptions and correct methods to assess at best the impact of uncertainty related to rotavirus epidemiology and estimation of quality of life in children aged under five years old on the cost-effectiveness ratios.

As most of economic models, one limitation of this study is that it relies on assumptions for some parameters which induce uncertainty around the estimates. Due to lack of evidence, data from nearby countries was used as proxy for Spain; this is the case for the percentage of working mothers (51%[39]), which is based on Italian data, or the percentage of cases not seeking for medical attention (41.4%[33]), based on French information. In both cases, differences in health behaviour within countries might be observed; for example, it is possible that more than 51% of mothers in Spain work although this parameter did not have any impact in the results. Secondly, we considered a coverage rate of 90% as indicated by the World Health Organization (WHO)[24] for common compulsory vaccinations. However, according to Martin A[53], average paediatric coverage rates might be higher in Spain (97%) due to higher reimbursement rates from the national healthcare payer compared with those in other European countries. This mainly implies that our results are certainly conservative and underestimate the real benefit of the vaccination.

Furthermore, the model did not consider additional indirect benefits due to rotavirus vaccination programme. For example, it is noteworthy that the epidemic peak of RVGE overlaps with that of other seasonal diseases such as influenza and respiratory syncytial diseases thus increasing the load for health services which are already overcrowded at this period[6]. By decreasing substantially the number of RVGE cases, the vaccination contributes to a better organisation of paediatric services at hospital as well as in PCCs.

Potential herd immunity resulting from transmission of vaccine strains has not been considered in this study. As rotavirus is transmitted by infants and children, it spreads within families and day care centres, the possibility of herd immunity would contribute substantially to the burden reduction. Preliminary data from the United States show that 2 years after the introduction of RV vaccination into the immunization schedule, the reduction in severe rotavirus disease appears to approximate that seen in phase III clinical trials. More over there also been reductions in rotavirus disease in older and unimmunised age groups[54,55]. In a recent study performed in five European countries where authors were evaluating cost -effectiveness of the rotavirus vaccination, the results show that incorporating the effect of possible indirect protection has overall a moderate impact on the cost-effectiveness ratio in all the countries. However, in some countries where the vaccination is not cost-effective but near to the threshold of 30.000€ per QALY the inclusion of indirect benefits of the vaccine could change the overall conclusion[56]. Although there have been reports of symptomatic cases due to transmission of vaccine rotavirus reported among family members, we did not take this into account due to the lack of available data. Generally, the limitations discussed would tend to lead to an underestimation of the vaccine benefits.

## Conclusions

In conclusion, this study shows that the implementation of a universal rotavirus vaccination program with RotaTeq would substantially reduce the morbidity due to RVGE among children in Spain, avoiding more than 136,200 cases and reducing spending by €38 million per birth cohort. Our model could be extended to assess the cost-effectiveness of a rotavirus vaccination program with RotaTeq in Spain.

## List of Abbreviations

AGE: Acute GastroEnteritis; DRG: Diagnostic Related Groups; ER: Emergency Room; GP: General Practioner; LOS: Length of Stay; NHS: National Health System; NNV: Number Needed to Vaccinate; OTC: Over The Counter; PCC: Primary Care Centre; REST: Rotavirus Efficacy and Safety Trial; REVEAL: Rotavirus gastroenteritis Epidemiology and Viral types Accounting for Losses in Public Health and Society; RVGE: Rotavirus GastroEnteritis; WHO: World Health Organization.

## Competing interests

Authors from IMS received fees from SPMSD to conduct the study

Authors from SPMSD has Non-financial competing interests (Commercial) being SPMSD employee

Diez-Domingo J. has received speaker fees from GSK and SPMSD, and is principal investigator in clinical trials and epidemiological studies financed by these companies. Sanofi Pasteur MSD give us grand support.

## Autors' contributions

JDD carried out the study, and participated in its design and coordination and helped to draft the manuscript, he read and approve the final manuscript. NLS carried out the study, and participated in its design and coordination and helped to draft the manuscript, she read and approve the final manuscript. NMA drafted the manuscript she read and approve the final manuscript. LB participated in the design of the study and the analysis and read and approve the final manuscript. NL initiated the study, participated in its coordination and helped to draft the manuscript, she read and approve the final manuscript. MT initiated the study, participated in its coordination and helped to draft the manuscript and read and approve the final manuscript.

## Pre-publication history

The pre-publication history for this paper can be accessed here:

http://www.biomedcentral.com/1471-2458/10/469/prepub
